# 2019 Audited schedule of changes in net assets

**DOI:** 10.5195/jmla.2020.1118

**Published:** 2020-10-01

**Authors:** BKD

**Affiliations:** 1 CPAs and Advisors, on behalf of the Medical Library Association, kelly.weaver@mci-group.com, Chicago, IL

The table below summarizes the association's financial status as of December 31, 2019. For a more complete audit report and related information, see the Audited Financial Statements 2001–2019 (members only). This report includes balance sheets, fund status reports, budgeted and actual revenues and expenditures, and a schedule of investments. Members may obtain a copy of the audit report from MLA headquarters.

**Table 1:** Medical Library Association schedule of changes in net assets by fund year ended December 31, 2019

	Net assets January 1, 2019	Contributions and other revenue	Invest-ment return	Net assets released from restrictions	Expenses	Net assets December 31, 2019
**Net assets without donor restrictions**						
General operating	($150,671)	$2,727,216	—	—	($3,003,435)	($426,890)
Other funds:						
Association stabilization	$1,688,760	—	$321,738	—	($39,409)	$1,971,089
Capital equipment	$2,228	—	—	—	—	$2,228
Special purpose	—	—	—	$124,228	($124,228)	—
Sections	$277,213	$41,150	—	—	($74,042)	$244,321
**Total net assets without donor restrictions**	$1,968,201	$41,150	$321,738	$124,228	($237,679)	$2,217,638
**Net assets with donor restrictions**						
Ysabel Bertolucci MLA Annual Meeting Grant Endowment	$31,774	$100	$7,425	($1,323)	—	$37,976
Estelle Brodman Award for the Academic Medical Librarian of the Year Endowment	$73,205	—	$7,274	($817)	—	$79,662
Naomi C. Broering Hispanic Heritage Grant Endowment	$27,743	$50	$6,308	($2,274)	—	$31,827
Lois Ann Colaianni Award for Excellence and Achievement in Hospital Librarianship Endowment	$26,818	—	$4,038	($676)	—	$30,180
Consumer Health Librarian of the Year Award Endowment	$25,898	—	$6,388	($1,278)	—	$31,008
Cunningham Memorial International Fellowship Endowment	$153,322	—	$30,346	($5,321)	—	$178,347
Louise Darling Medal for Distinguished Achievement in Collection Development in the Health Sciences Endowment	$82,350	—	$8,918	($1,388)	—	$89,880
Janet Doe Lectureship Endowment	$56,914	$300	$6,549	($785)	—	$62,978
Carla J. Funk Governmental Relations Award Endowment	$19,884	$1,442	$4,168	($180)	—	$25,314
Eugene Garfield Research Fellowship Endowment	$132,075	$166	$30,847	($1,342)	—	$161,746
T. Mark Hodges International Service Award Endowment	$4,656	—	$1,224	($53)	—	$5,827
Hospital Libraries Section/MLA Professional Development Grant Endowment	$48,230	—	$6,899	($693)	—	$54,436
David A. Kronick Traveling Fellowship Endowment	$19,923	—	$8,079	($352)	—	$27,650
Joseph Leiter NLM/MLA Lectureship Endowment	$78,433	—	$10,499	($10,457)	—	$78,475
Librarians without Borders® Ursula Poland International Scholarship Endowment	$27,232	—	$5,749	($1,250)	—	$31,731
Donald A. B. Lindberg Research Fellowship Endowment	$302,139	$5,300	$54,376	($12,366)	—	$349,449
Majors/MLA Chapter Project of the Year Endowment	$18,583	—	$2,836	($623)	—	$20,796
Lucretia W. McClure MLA Excellence in Education Award Endowment	$53,656	$980	$9,250	($903)	—	$62,983
John P. McGovern Award Lectureship Endowment	$126,951	$500	$28,624	($6,246)	—	$149,829
MLA Disaster Relief Fund	$6,354	—	—	—	—	$6,354
Scholarship Endowment	$253,915	$16,870	$85,975	($12,274)	—	$344,486
Section Project of the Year Award Endowment	($642)	—	—	($500)	—	($1,142)
Shaping Our Future Endowment	$69,920	$1,600	$12,971	($564)	—	$83,927
Special Purpose/Librarians without Borders®	$139,081	—	—	($40,239)	—	$98,842
Special Purpose/MLA/NLM Spectrum Scholarships	$26,000	—	—	($22,324)	—	$3,676
**Total net assets with donor restrictions**	$1,804,414	$27,308	$338,743	($124,228)	—	$2,046,237
**Total all net assets**	$3,621,944	$2,795,674	$660,481	—	($3,241,114)	$3,836,985

